# Developing a Disease Outbreak Event Corpus

**DOI:** 10.2196/jmir.1323

**Published:** 2010-09-28

**Authors:** Mike Conway, Ai Kawazoe, Hutchatai Chanlekha, Nigel Collier

**Affiliations:** ^2^Center for Women in ResearchTsuda CollegeTokyoJapan; ^1^National Institute of InformaticsTokyoJapan

**Keywords:** Biosurveillance, disease outbreaks, natural language processing, corpora, text mining, information extraction, public health informatics

## Abstract

**Background:**

In recent years, there has been a growth in work on the use of information extraction technologies for tracking disease outbreaks from online news texts, yet publicly available evaluation standards (and associated resources) for this new area of research have been noticeably lacking.

**Objective:**

This study seeks to create a “gold standard” data set against which to test how accurately disease outbreak information extraction systems can identify the semantics of disease outbreak events. Additionally, we hope that the provision of an annotation scheme (and associated corpus) to the community will encourage open evaluation in this new and growing application area.

**Methods:**

We developed an annotation scheme for identifying infectious disease outbreak events in news texts. An event─in the context of our annotation scheme─consists minimally of geographical (eg, country and province) and disease name information. However, the scheme also allows for the rich encoding of other domain salient concepts (eg, international travel, species, and food contamination).

**Results:**

The work resulted in a 200-document corpus of event-annotated disease outbreak reports that can be used to evaluate the accuracy of event detection algorithms (in this case, for the BioCaster biosurveillance online news information extraction system). In the 200 documents, 394 distinct events were identified (mean 1.97 events per document, range 0-25 events per document). We also provide a download script and graphical user interface (GUI)-based event browsing software to facilitate corpus exploration.

**Conclusion:**

In summary, we present an annotation scheme and corpus that can be used in the evaluation of disease outbreak event extraction algorithms. The annotation scheme and corpus were designed both with the particular evaluation requirements of the BioCaster system in mind as well as the wider need for further evaluation resources in this growing research area.

## Introduction

The need for computational tools for the tracking of emerging disease outbreaks from text has become increasingly important in recent years [1,2] leading to the development of various machine-aided surveillance systems (eg, Global Public Health Intelligence Network (GPHIN) [3], HealthMap [4], BioCaster [5], MedISys [6], Pattern-based Understanding and Learning System (PULS) [7], and EpiSPIDER[8]). One way to evaluate the semantics of such a system is to construct an event frame (ie, template), which is then associated with each outbreak event in a sample of news documents (the nature and scope of reportable events varies according to the case definition of each system). This paper reports on such a data set─an annotation scheme and corpus [9]─developed for disease outbreak event detection in the context of the BioCaster biosurvellance online news information extraction (IE) system [10,5].

We believe that a focus on event extraction offers additional advantages to methods based solely on information retrieval (IR). Traditional IR systems allow us to identify reports based on the presence or absence of disease terms whereas event-based IE approaches enable us to dig deep into a report’s semantics. The mere presence of a disease term in a text should not necessarily lead us to the conclusion that the report contains pressing information about an outbreak. Indeed, Steinberger et al estimated that 63% of documents selected using traditional IR techniques do not contain outbreak events [11]. For example, vaccination campaigns, medical research results, and public health advice often occur in news texts and are likely to generate false positives if we rely solely on IR to identify documents of interest. An event-based strategy facilitates the exclusion of nonrelevant documents from further processing and could form the basis of more sophisticated text mining and visualization while providing richer outbreak data for end users. Note that the event-based approach suggested here requires antecedent document selection and named entity recognition (NER) modules (ie, a pipeline with a document selection module inputting relevant documents to an NER module before this output is piped to an event extraction module). In the case of the BioCaster system, the document selection module has a particularly important “gate-keeping” function as the system accepts input from over 1700 RSS feeds─far too many documents to subject to the computationally intensive NER and event extraction processes [10].

The event annotation scheme aims to identify each infectious disease outbreak event in a given text with its associated disease, time, location (at various levels of granularity), and other relevant information. An annotated corpus is necessary in order to evaluate the performance of the current BioCaster IE system and also serves as a test bed for the development of new biosurveillance-specific IE algorithms and techniques. Further, the provision of a reusable resource facilitates further work on disease event extraction and encourages the development of the field, as it has been shown that the provision of such resources (often in conjunction with organized “challenge evaluations” similar to, for instance, the Text Retrieval Conference (TREC) Genomics Track [12]) has increased research momentum for other IE tasks [13].

Previous work on evaluation for disease outbreak report IE systems has focused on disparate aspects of performance. For example, Blench [14] found that the GPHIN system identified 56% of the outbreaks verified by the World Health Organization (WHO) over a three-year period, while Freifeld et al [15] found that the HealthMap system successfully classified 84% of reports by disease and location over a one-month period. Kawazoe et al [16] reported that the BioCaster system’s NER module achieved an F-score of 76.97, while for the PULS system (which is an event extraction system that relies on input from the MedISys IR system), it is estimated that approximately 72% of the extracted events are correct [11]. While this kind of evaluation work is important for system developers, the obvious difficulty in comparing reported results illustrates the need for a community-wide data set for algorithm testing.

The structure of the paper is as follows. First, we describe the event annotation scheme we developed, then, we set out agreement statistics before finally presenting a description of the corpus and associated software.

## Annotation Scheme

**Figure 1:**
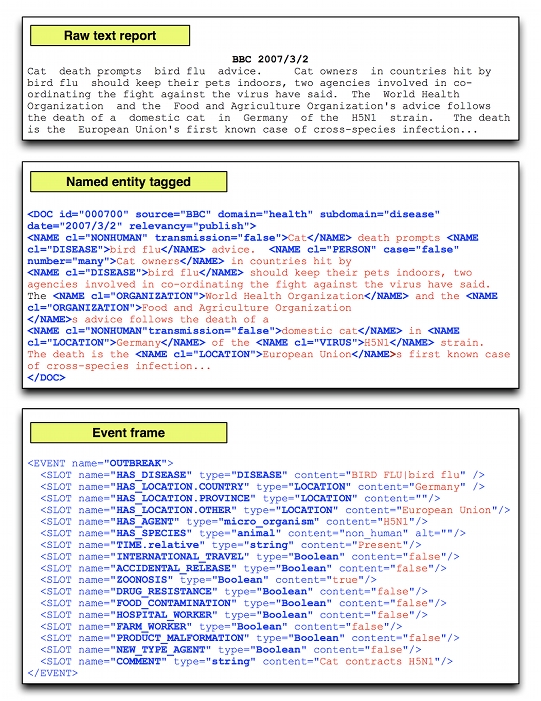
Worked example of event frame construction from raw text. Note that this paper focuses on the construction of event frames from documents already tagged for named entities. The named entity tagging process is described by Kawazoe et al [16].

Each document is associated with zero or more event frames reflecting the number of outbreak events described in the text (A full description of the annotation scheme, and all associated software can be downloaded from the project Google Code site [9]). The event frames are designed to capture the properties of outbreak reports that are of interest to public health experts and epidemiologists. Event frames are formatted in extensible markup language (XML) (see Figure 1) and consist of property names and their associated values derived from the document source (eg, HAS_DISEASE, “Ebola”). Reports have already been tagged for named entities such as person names, disease names, location names, and so on (twelve in total) using an ontology-based annotation scheme developed specifically for the disease outbreak domain [16]. Property names are of two types. First, entity properties are filled with appropriate entities derived directly from the text of interest (entity properties are conceptually similar to Message Understanding Conference (MUC) style “string fills”). For example, the HAS_DISEASE property could only have the value “polio” if “polio” is tagged as an entity in the document. Second, fixed slots (equivalent to MUC-style “set fills”) take prespecified values of a restricted kind (normally simply Boolean true or false values), and, unlike entity values, are *inferred* from the document. For example, the INTERNATIONAL_TRAVEL property accepts only Boolean values.

The following are the entity properties (which are filled by named entities) and their definitions:

- HAS_DISEASE: disease that caused the outbreak (eg, Ebola)
- HAS_LOCATION.COUNTRY: country where the outbreak occurred (eg, United States, Indonesia)
- HAS_LOCATION.PROVINCE: province in which the outbreak occurred (eg, Kanagawa, New Hampshire)
- HAS_LOCATION.OTHER: other geographical location (eg, Balkans, New England)
- HAS_AGENT: agent (pathogen) of the disease (eg, HIV)

The following are the “fixed” slots (which are inferred from the text and take prespecified values) and their definitions:

- HAS_SPECIES: human or non_human
- TIME.relative: historical (more than three months ago), recent_past (between two weeks and three months ago), present (within the last two weeks), and hypothetical

- ZOONOSIS: has species transfer occurred? (Boolean)
- DRUG_RESISTANCE: is the disease drug resistant? (Boolean)
- NEW_TYPE_AGENT: is the disease a new strain? (Boolean)
- ACCIDENTAL_RELEASE: has the disease been released accidently? (Boolean)
- INTERNATIONAL_TRAVEL: is international travel involved? (Boolean)
- FOOD_CONTAMINATION: is the outbreak caused by contaminated food or water? (Boolean)
- HOSPITAL_WORKER: are any victims hospital workers? (Boolean)
- FARM_WORKER: are any victims farm workers? (Boolean)
- MALFORMED_PRODUCT: are contaminated blood products or vaccines implicated? (Boolean)

A working group consisting of the current paper’s authors developed the annotation scheme over a period of several months guided by the World Health Organization International Health Regulations [17] (see Textbox 1) and using advice provided by the National Institute of Infectious Diseases in Japan.

**Using the World Health Organization international health regulations (Annex 2 decision instrument) as the basis for an annotation scheme**

**World Health Organization International Health Regulations (Annex 2 Decision Instrument)**

In developing our guidelines, we took inspiration from the World Health Organization's International Health Regulations Annex 2 decision instrument [17] for building our own decision tree. At the top level, this included general questions such as, "Is the topic of the article mainly about a current disease outbreak?" and "Are the victims of the disease mainly humans?" These questions were designed to guide annotators in the most obvious stages of selection. At lower levels, the decision tree we developed touched on issues more central to the Annex 2 decision tree such as known notifiable diseases (eg, SARS, Smallpox, Poliomyelitis, Cholera, West Nile Virus). The notion of “unusual” or “unexpected” is underspecified in Annex 2 but would be apparent to a public health expert familiar with the field. We tried to make the notion explicit for our annotators by encoding questions about the virulence and infectivity of the pathogen, the severity of the reported cases, the involvement of international travel, and drug resistance or accidental/deliberate release.

## Agreement Study and Error Analysis

To gain insight into how consistently the scheme could be applied and to help pinpoint areas of systematic annotator error, we conducted a 100-document interannotator agreement study. We recruited and trained one annotator and compared that individual’s annotations with those of an annotator who was involved in the original annotation scheme design process.

Following the recommendations of Wilbur et al [18], we used percentage scores to assess agreement rather than the kappa statistic [19]. While some researchers in annotation scheme design refrain from the use of agreement studies entirely (eg, [20]), we felt that this exercise would help to draw out any systematic annotator difficulties and also facilitate the debugging of the annotation scheme and corpus.

We found that the two annotators agreed on the number of disease outbreak events 67% of the time. However, calculating agreement at the level of individual properties (eg, TIME.relative) was not as straightforward as calculating event number agreement for the following three reasons: (1) Annotators could identify a differing number of events for a document. (2) Unless both annotators produced just 1 (or zero) event frames, we were faced with the problem of aligning events. (3) The annotation scheme allowed for an arbitrary number of property values, reflecting synonymous or near synonymous terms in the source document. For example, it was not unusual to see a property/value pairing such as HAS_DISEASE=“bird flu|H5N1|avian influenza.”

Therefore, we concentrated our analysis on those 42 documents where only one event was identified per annotator, thus allowing for a direct comparison. These data are partially summarized in Table 1, where it can be seen that the annotators agreed 100% of the time on DRUG_RESISTANT, FARM_WORKER, INTERNATIONAL_TRAVEL, and PRODUCT_MALFORMATION. Agreement was worst for FOOD_CONTAMINATION and ZOONOSIS. Major sources of disagreement are summarized in Textbox 2.

The fixed slot properties, TIME.relative and SPECIES, are not Boolean and therefore are not represented in Table 1. TIME.relative had four values (historical, recent past, present, and hypothetical) and achieved an agreement score of 90.5% (with the most frequent value being “present”). SPECIES had two values (human and nonhuman) and achieved an agreement of 90.2%. More information about the annotation guidelines is available in [9].

The entity properties (eg, HAS_LOCATION.PROVINCE, HAS_DISEASE) were filled by tagged entities in the text. Agreement for HAS_DISEASE was 100% and for HAS_LOCATION.COUNTRY was 97.7%.

**Table 1:** Agreement for 42 documents with precisely one event per annotator (note that only Boolean fixed slot properties are shown)

	Agreement for Fixed Slot Properties in Each of 42 Documents				
Property	Annotator 1 (true)	Annotator 1 (false)	Annotator 2 (true)	Annotator 2 (false)	Agreement (%)
DRUG_RESISTANCE	0	42	0	42	100.0
FARM_WORKER	0	42	0	42	100.0
FOOD_CONTAMINATION	5	37	13	29	71.4
HOSPITAL_WORKER	0	42	1	41	97.6
INTERNATIONAL_TRAVEL	0	42	0	42	100.0
PRODUCT_MALFORMATION	0	42	0	42	100.0
ZOONOSIS	7	35	12	30	83.0

**Sources of disagreement**

**Event Agreement**

On detailed examination of the data, a systematic problem concerning event granularity emerged accounting for the relatively low 67% event agreement rate. Our analysis showed that the issue of suspected zoonosis (ie, unconfirmed zoonosis or where zoonosis is presented as one possible explanation for a human disease) was central here. One annotator produced two events (one human, one non_human), while the other annotator only produced one event (human), ignoring the suspicion of, or speculation about, zoonosis.

**Annotator Error**

We can distinguish annotator agreement arising from ambiguity in the annotation guidelines from straightforward annotator mistakes. For instance, there are several examples where the temporal categories, present (within two weeks of the document time stamp) and recent_past (more than two weeks, but less than three months from the document time stamp), were confused.

**Background Knowledge and Inference**

For those properties that require an annotator to infer a category from the document (TIME.relative, ZOONOSIS, HAS_SPECIES, INTERNATIONAL_TRAVEL, DRUG_RESISTANCE, FOOD_CONTAMINATION, HOSPITAL_WORKER, FARM_WORKER, and PRODUCT_MALFORMATION), there is scope for incorrect inference. For example, several of the documents in the agreement study data set concern cholera. While cholera is spread primarily through water contamination (ie, FOOD_CONTAMINATION), this is not stated explicitly in the text. Only one of the annotators marked these documents as true for FOOD_CONTAMINATION, suggesting that the annotator who marked the property false was unaware of the primary transmission route for cholera.

## Corpus Description

The corpus consists of 200 documents (all in English) and their associated event frames, with documents gathered from a variety of sources (see Table 2). The largest single source was ProMed-Mail [21], an expert-curated infectious disease reporting service. Additionally, documents were not randomly sampled, but rather selected to represent diseases and geographical areas of interest to the researchers. Major international news providers are also represented (eg, CBC, Reuters, BBC) in addition to primarily Asian or Asia-Pacific news services (eg, Vietnam-net, Thailand’s The Nation). Documents range from 45 to 1487 words long, with a mean of 305.9 words (without markup). Document selection was performed by author MC (see corpus documentation [9] for details).

**Table 2:** Corpus document sources (200 documents)

Document Source	Number of Documents	% of 200
ProMed-Mail	43	21.5
Reuters	16	8.0
BBC	16	8.0
WHO	41	20.5
CBS	13	6.5
CBC	17	8.5
Vietnam-net	12	6.0
Hindustan Times	18	9.0
The Nation (Thailand)	9	4.5
All Africa	5	2.5
Xinhua (China)	5	2.5
Antara (Indonesia)	5	2.5

**Figure 2:**
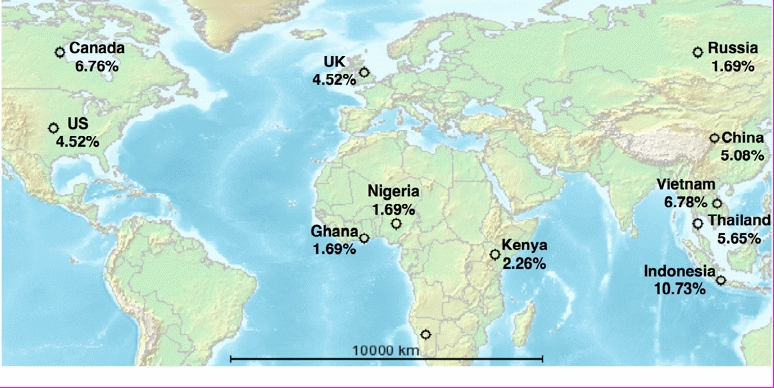
Distribution of disease events in our corpus by country (only countries with 2 or more events shown) (Map produced by GPS visualizer)

Of the 394 annotated events in the corpus, 75.4% describe human (rather than animal) disease events (see Table 3). Most of the events identified (81.5%) have been classified as present outbreaks, although historical, recent past, and hypothetical events are also represented. To show the geographical range of the documents selected, the geographical distribution of events (by country) is shown in Figure 2. Note that the map does not show the actual distribution of disease events, but rather the geographical distribution of disease events in our corpus*.*

**Table 3:** Event statistics (total number of events is 394)

Type of Event	Number of Events	% of 394
Events involving humans	297	75.4
Events involving food contamination	35	8.9
Events involving hospital workers	3	0.8
Events involving malformed products	2	0.5
Events classified as present	321	81.5
Events classified as historical	49	12.4
Events classified as recent_past	11	2.8
Events classified as hypothetical	13	3.3

**Figure 3:**
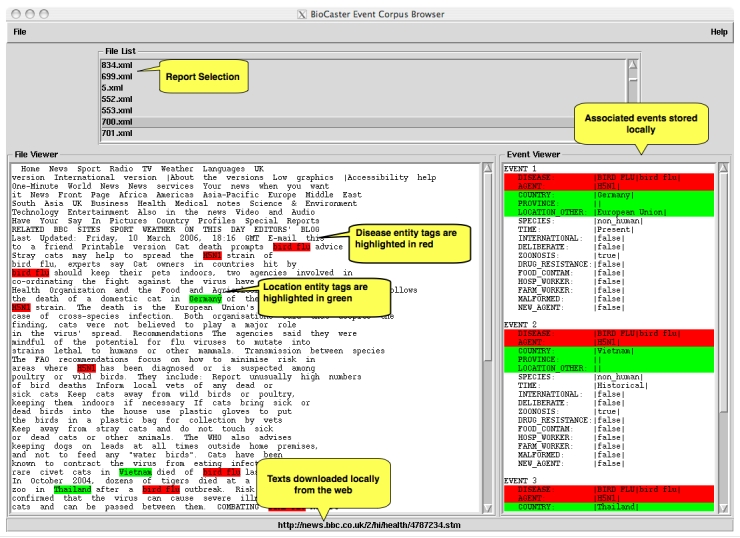
Linux BioCaster corpus event frame browsing tool [9]

While we hope that the event frame may form part of the foundation for a future standard, we recognize that there are challenges in achieving this goal (see Textbox 3). Further, due to copyright restrictions, we are unable to distribute the corpus directly. Instead we have provided two methods for corpus access. First, a download script (a Perl script that downloads and cleans all the documents from their original source on the Web and then associates them with event frames) and a graphical user interface (GUI) based event browser (see Figure 3) [9]. Note that as of July 2009, only 176 of the original 200 documents were currently available online.

**Barriers to general adoption of the event annotation scheme**

**Heterogeneous Systems and Requirements**

The current event frames may not be suitable for all needs. For some users, the knowledge required by event frames may be superfluous (eg, a system that is solely concerned with identifying cholera outbreak has no need for zoonosis information). For other users, the event frame may not encode enough information (eg, an event's certainty or uncertainty—unrepresented in our event frame—may be important for system designers). Indeed, it is conceivable that some users may suffer from both these problems. Nevertheless, we believe that our event scheme provides a foundation for potential future standards developments.

**Agreement**

The current agreement level for number of events (67%) is not high. However, this result masks the fact that agreement for important entity properties such as HAS_LOCATION.COUNTRY and HAS_DISEASE is almost perfect.

**Extending to New Genres**

The current scheme was designed for news text. It is not clear how well the scheme would extend to other, less formal genres that may contain information of interest (eg, blog postings and message boards).

In summary, we present an annotation scheme and corpus that can be used in the evaluation of disease outbreak event extraction algorithms. The annotation scheme and corpus are presented to the research community in the belief that such resources can help in the formation of an emerging standard for this rapidly growing research area.

## Abbreviations

GPHIN: Global Public Health Intelligence Network
GUI: graphical user interface
IE: information extraction
IR: information retrieval
MUC: message understanding conference
NER: named entity recognition
PULS: Pattern-based Understanding and Learning System
TREC: Text Retrieval Conference
WHO: World Health Organization
XML: extensible markup language
